# Adaptive immune cells and kidney function in older adults: sex-specific associations in the health and retirement study (HRS)

**DOI:** 10.3389/fragi.2026.1882462

**Published:** 2026-08-25

**Authors:** Shannon M. Sullivan, Bibin Joseph, Shweta Sharma, Weihua Guan, Amy B. Karger, Jessica Faul, Bharat Thyagarajan

**Affiliations:** 1 Department of Laboratory Medicine and Pathology, University of Minnesota, Minneapolis, MN, United States; 2 Division of Epidemiology, School of Public Health, University of Minnesota, Minneapolis, MN, United States; 3 Division of Biostatistics and Health Data Sciences, School of Public Health, University of Minnesota, Minneapolis, MN, United States; 4 Survey Research Center, Institute for Social Research, University of Michigan, Ann Arbor, MI, United States

**Keywords:** CD8 T cells, chronic kidney disease, estimated glomerular filtration rate, immune cells, kidney function, sex differences, T cells, B cells

## Abstract

**Background:**

Chronic kidney disease (CKD) is characterized by chronic inflammation and immune dysregulation. Aging of the adaptive immune system, marked by a shift from naïve to memory T-cell phenotypes, is associated with systemic “inflammaging”, but sex-specific associations between these immunophenotypes and kidney function in older adults remain poorly understood.

**Methods:**

We analyzed cross-sectional data from 8,970 participants (aged ≥56 years) in the 2016 Health and Retirement Study (HRS) Venous Blood Study. Seventeen adaptive immune cell subsets were quantified using multiparameter flow cytometry. Kidney function was assessed using estimated glomerular filtration rate (eGFR, mL/min/1.73 m^2^) calculated from the 2021 race-free CKD-EPI creatinine-cystatin C equation. Survey-weighted linear regression models were adjusted for age, race/ethnicity, education, body-mass index (BMI), clinical comorbidities (diabetes, elevated blood pressure/hypertension), alcohol use, smoking status, and cytomegalovirus (CMV) serostatus. Sex-stratified analyses were conducted *a priori*, and sex interaction terms were evaluated to assess effect modification.

**Results:**

Females were older and more likely to be never smokers, CMV seropositive, and have lower eGFR, whereas males were more likely to use alcohol, smoke, have diabetes, elevated blood pressure/hypertension and higher educational attainment. In sex-stratified models, higher CD4^+^ T cells (β = 0.96, P_FDR_ = 0.010), CD8^+^ naïve T cells (β = 0.87, P_FDR_ = 0.026), Ratio of CD4^+^ to CD8^+^ T cells (β = 0.73, P_FDR_ = 0.036), and lymphocytes (β = 2.42, P_FDR_<0.001) were associated with higher eGFR (higher kidney function) in females, while higher CD4^+^ effector memory T cells (Tem; β = −1.58, P_FDR_ = 0.010), CD8^+^ effector T cells (Teff; β = −1.00, P_FDR_ = 0.004), and CD8^+^ Tem (β = −1.40, P_FDR_ = 0.002) were associated with lower eGFR (lower kidney function). In males, higher IgD + memory B cells (β = 1.01, P_FDR_ = 0.005), IgD− memory B cells (β = 1.04, P_FDR_ = 0.011), and lymphocytes (β = 2.08, P_FDR_<0.001) were associated with higher eGFR. Significant sex interaction was observed for CD8^+^ Tem (P_FDR_ = 0.015), with a nominal interaction for CD8^+^ Teff (P_FDR_ = 0.060). Associations were similar in sensitivity analyses and were not modified by CMV serostatus.

**Conclusion:**

Findings suggest that CD8^+^ Tem and Teff T cells may serve as sex-specific biomarkers of renal vulnerability in postmenopausal females and underscore the need for longitudinal and mechanistic studies to clarify underlying pathways.

## Introduction

Chronic kidney disease (CKD) is a common and progressive condition affecting more than 10% of the global population and approximately 15% of adults in the United States (Kovesdy, 2022; United States Renal Data System, 2023; Ortiz et al., 2026). Its prevalence increases with age and is strongly influenced by diabetes, hypertension, and obesity (United States Renal Data System, 2023; Mark et al., 2025). As one of the leading causes of morbidity and mortality worldwide, CKD is among one of the few non-communicable diseases that show a significant increase in associated deaths over the past 2 decades (Kovesdy, 2022). Therefore, identifying biological pathways that contribute to kidney function decline in older adults remains an important public health priority.

A growing body of evidence suggests that CKD is not merely a renal disorder but also a systemic condition characterized by chronic inflammation and immune dysregulation (Tecklenborg et al., 2018; Wu et al., 2022; Espi et al., 2020; Liakopoulos et al., 2017). Both aging and CKD are associated with shifts in adaptive immunity characterized by loss of naïve T cells, expansion of memory, effector, and highly differentiated (Tem/Teff) T cell phenotypes, and reduced thymic output (Wu et al., 2022; Márquez et al., 2020; Lioulios et al., 2026; Sopena-Rios et al., 2026; Chi et al., 2022; Winterberg and Ford, 2017; Litjens et al., 2024; Elmenyawi A et al., 2022; Chen et al., 2025; Hartzell et al., 2020; Chiu et al., 2018; Iio et al., 2023; Song et al., 2026; Be et al., 2012; Betjes et al., 2011). These shared features are often described as “immune aging” or immunosenescence and may reflect chronological aging or disease-related processes such as uremia, oxidative stress, and chronic inflammation in CKD (Tecklenborg et al., 2018; Wu et al., 2022; Espi et al., 2020; Liakopoulos et al., 2017; Winterberg and Ford, 2017; Litjens et al., 2024; Elmenyawi A et al., 2022; Chen et al., 2025; Hartzell et al., 2020; Chiu et al., 2018; Iio et al., 2023; Song et al., 2026; Be et al., 2012). In advanced kidney disease and end stage renal disease (ESRD), pronounced naïve T cell depletion, reduced recent thymic emigrants, and accumulation of highly differentiated CD4^+^ and CD8^+^ T cells have been well described (Winterberg and Ford, 2017; Litjens et al., 2024; Elmenyawi A et al., 2022), but it remains unclear whether similar immune changes are present earlier in the course of declining kidney function.

Sex differences in these immune-aging patterns have been reported in older adults, with females generally retaining stronger adaptive and B cell responses, whereas males exhibit more pronounced age-related increases in cytotoxic and monocyte-driven inflammatory activity, particularly after age 65 (Márquez et al., 2020; Sopena-Rios et al., 2026; Liao et al., 2026; Layug et al., 2024; Klein and Flanagan, 2016; Gheitasi et al., 2025; Kim et al., 2021; Huang et al., 2021; Robinson et al., 2022). Clinically, females having a higher prevalence of CKD but males tend to progress faster and experience worse outcomes once CKD is established (Kattah and Garovic, 2020; Melsom et al., 2022; Vosters et al., 2024). These immune sex differences intensify with age, yet the extent to which sex-biased immune aging contributes to differences in kidney function remains unclear (Márquez et al., 2020; Kattah and Garovic, 2020; Melsom et al., 2022; Vosters et al., 2024). Prior studies in both aging cohorts and clinical CKD populations suggest that lower CD4/CD8 ratios and altered T cell homeostasis are associated with impaired kidney function (Chen et al., 2025; Hartzell et al., 2020). In studies of participants with established kidney failure, T cell exhaustion, chronic activation, and loss of naïve T cells characterize advanced uremia, and these immune changes have been interpreted as part of CKD-driven immunosenescence (Winterberg and Ford, 2017; Litjens et al., 2024; Elmenyawi A et al., 2022). However, despite these sex-specific patterns, most existing evidence derives from patients with advanced CKD or ESRD, where immune dysfunction is already pronounced, making it difficult to determine whether immune alterations precede CKD onset or arise as a consequence of declining kidney function. Also, few studies have explicitly examined sex differences, despite the well-known sex-specific patterns in both immune aging and CKD epidemiology.

To address these knowledge gap, we used multiparameter flow cytometry measures from the Health and Retirement Study (HRS) 2016 Venous Blood Study (VBS) (Health and Retirement Study, 2025) to examine cross-sectional associations between adaptive immune cell subsets and estimated glomerular filtration rate (eGFR) in older adults, with a focus on sex-specific differences. We evaluated adaptive immune cell subsets including CD4^+^ and CD8^+^ T cell subsets (naïve, central memory, effector, and effector memory), the CD4/CD8 ratio, B cell subsets (incluing IgD+ and IgD-memory and naïve), and total lymphocytes to better characterize how immune aging phenotypes may relate to kidney function in later life.

## Materials and methods

### Data source

HRS is an on-going bi-annual nationally representative longitudinal survey of more than 50,000 individuals over the age of 50 in the United States designed to examine health, economic, and social factors affecting an aging population. The study is sponsored by the National Institute on Aging (NIA) and is conducted by the University of Michigan’s Institute for Social Research.

### HRS 2016 venous blood study (VBS) sample

Panel respondents who completed the 2016 wave interview were invited to participate in a venous blood draw as part of the VBS, as documented previously (Crimmins et al., 2017). The final VBS sample included 9,934 participants (65% final completion among eligible respondents). The consent rate for the VBS component of the survey was 78.5%. Of these, 82.9% completed collection for a final completion rate of 65% among eligible participants resulting in a sample size of 9,932 with extensive blood-based phenotype data along with survey-based demographic and social data.

### Outcome–kidney function

Kidney function was assessed using estimated glomerular filtration rate (eGFR) calculated with the 2021 CKD-EPI Creatinine-Cystatin Equation (race-free), using serum biomarkers of creatinine (measured with the enzymatic creatinine method on the Roche Cobas 6,000) and cystatin C (measured with the immunoturbidometric Gentian method on the Roche Cobas 6,000), age, and sex (Crimmins et al., 2017; Inker et al., 2021). eGFR was modeled as a continuous outcome to capture variation in kidney function across the sample.

### Exposure–adaptive immunophenotyping

Peripheral blood mononuclear cells (PBMCs) were isolated from whole blood and used to measure 17 adaptive immune cell subsets on a LSRII flow cytometer or a Fortessa X20 instrument (BD Biosciences, San Diego, CA) (Barcelo et al., 2018). While the HRS VBS measured 29 immune cell subsets (Health and Retirement Study, 2021), this analysis focused on the adaptive immune compartment, including 11 T cells CD4/CD8 ratio, 4 B cell subsets, and total lymphocytes. Immunophenotyping data were analyzed using OpenCyto and FlowAnnotator via hierarchical gating strategies as described previously, with additional refinement conducted in FlowJo software (FlowJo LLC version 10, Ashland, OR) when necessary (Hunter-Schlichting et al., 2020). Detailed laboratory methods, subset definitions, and gating strategies used in HRS have been described in detail in prior publications (Hunter-Schlichting et al., 2020; Thya et al., 2018; Thyagarajan et al., 2022).

Markers used to define adaptive immune cells (T, B, and lymphocyte subsets) are listed in Supplementary Table S1. In this project, immune cell subsets were expressed as the percentage (%) of their parent population (e.g., CD8^+^ effector memory T cells as a percentage of total CD8^+^ T cells). The CD4+/CD8+ T cell ratio was calculated as a measure of systemic immune aging and homeostasis.

### Other covariates

Demographic and lifestyle factors were obtained from the HRS core survey, including participants’ age at venous blood collection, self-reported sex (female/male), race/ethnicity (Non-Hispanic White/Caucasian, Non-Hispanic Black/African American, Hispanic, and Non-Hispanic other race/ethnicity), education (<highschool, highschool/general education development [GED], some college, college/professional degree), alcohol use (yes/no), and smoking status (current, former, never). Body-mass index (BMI) was calculated as weight (pounds) divided by height squared (inches^2^) multiplied by 703, prioritizing interviewer-measured weight and height; if unavailable, self-reported values from the 2016 or 2014 surveys waves (collected every 4 years) were used.

Clinical comorbidities were defined using a combination of objective biomarkers and self-reported data from the 2016 or 2014 surveys waves. Elevated blood pressure/hypertension was defined as systolic blood pressure ≥ 120 mmHg, diastolic blood pressure of ≥ 80 mmHg, self-reported use of antihypertensive medications, or any combination of these. Diabetes status was defined as HbA1c ≥ 6.5%, a fasting plasma glucose of ≥ 126 mg/dL, or self-reported physician diagnosis plus use of oral hypoglycemic medication or insulin; pre-diabetes was defined as HbA1c 5.7%–6.4% or fasting plasma glucose 100–125 mg/dL. Cytomegalovirus (CMV) serostatus, a critical factor in immune aging, was assessed using Elecsys CMV IgG electrochemiluminescence immunoassay (Model e411; Roche Diagnostics) using the serum ratio of total immunoglobulin G (IgG) to anti-CMV IgG (Health and Retirement Study, 2025).

### Statistical analysis

The final analytical sample consisted of 8,970 HRS participants with valid survey weights, complete data on eGFR and all primary covariates, and data available for at least one adaptive immune cell subset (Supplementary Figure S1). From the initial HRS 2016 VBS Study (n = 9,934), we excluded participants with non-positive survey weights (PVBSWGTR; n = 717) and incomplete data for serum biomarkers required for eGFR calculation (n = 80), race/ethnicity (n = 6), education (n = 3), BMI (n = 69), CMV serostatus (n = 5), or immunophenotyping data (n = 84). Analyses of individual immune cell subsets were conducted using all participants with non-missing data for the corresponding subset. Sample sizes for each immune cell subset are detailed in Supplementary Table S2. Participants missing a specific immune cell subset were excluded only from analyses involving that subset and were retained in all other eligible analyses; no imputation was performed.

This analysis focused on 17 adaptive immune cell subsets: 16 measured subsets (11 T cells, 4 B cells, and total lymphocytes) and the calculated CD4+/CD8+ T cell ratio. Sex-based differences in demographics and clinical variables were tested using chi-squared tests for categorical variables and two-sample t-test for continuous variables.

Immune cell proportions were right-skewed, so subsets were natural-log transformed. To facilitate comparison of effect estimates across immune cell subsets, log-transformed values were divided by the standard deviation (SD) of the log-transformed distribution in the overall analytic sample; SDs used for scaling are shown in Supplementary Table S2. Effect estimates (betas) are therefore reported per one-SD change in log-transformed immune cell. Given established sex differences in kidney disease and immune cells, sex-stratified analyses (female and male, separately) were specified *a priori* as the primary analytic approach. Survey-weighted linear regression models estimated the association between each standardized, log-transformed immune cell subset and eGFR, adjusted for age, sex (where appropriate), race/ethnicity, education, BMI, blood pressure status, diabetes status, alcohol use, smoking status, and CMV serostatus (Model 1). To formally test for effect modification by sex, an overall (non-stratified) model including a sex-by-immune cell interaction term was also estimated, adjusted for the same covariates as Model 1 + sex (female/male). Since circulating inflammatory markers may lie downstream of adaptive immune cell activation and could therefore represent either confounders or mediators of the immune cell-eGFR relationship, our primary models did not adjust for systemic inflammation. To assess the extent to which observed associations were independent of systemic inflammation, we conducted a sensitivity analysis (Model 2) additionally adjusting for an inflammatory latent class variable derived by the HRS team from five log-transformed inflammatory biomarkers (C-reactive protein, interleukin-6, interleukin-10, interleukin-1 receptor antagonist, and soluble tumor necrosis factor receptor 1) (Meier et al., 2023). Sensitivity analyses were also repeated after excluding participants with eGFR<60 mL/min/1.73 m^2^ and eGFR<30 mL/min/1.73 m^2^ to ensure observed associations were robust and not primarily driven by individuals with existing moderate-to-severe kidney dysfunction (CKD Evaluation and Management–KDIGO, 2025). All models incorporated HRS VBS survey weights (pvbswgtr) and design effect variables (strata and cluster) to obtain an estimate representative of the U.S. population aged 56 years and older (Collins et al., 2026). False discovery rate correction (Benjamini–Hochberg) was applied separately across the 17 immune cell subsets evaluated within each analytic framework (sex-stratified, overall, and sensitivity analyses). All analyses were conducted in STATA and figures were generated in R (version 4.4.1) (R Core Team, 2021; Benjamini and Hochberg, 1995).

## Results

The unweighted analytic sample (n) included 8,970 HRS participants aged 56 years and older, including 53.9% females (n = 5,159) and 46.1% males (n = 3,811; Table 1). Females were older than males (mean age 69.0 vs. 67.9 years), had a higher prevalence of CMV seropositivity (68.7% vs. 57.3%), and were more likely to be never smokers (51.3% vs. 37.6%). Males had a higher prevalence of alcohol use (66.1% vs. 55.1%), be current smokers (12.4% vs. 10.0%), former smokers (50.0% vs. 38.7%), have diabetes (23.2% vs. 20.4%), and have a college/professional education (34.8% vs. 26.3%). Females also had a higher prevalence of kidney disease, defined as eGFR<60 mL/min/1.73 m^2^ (24.0% vs. 19.1%). Mean BMI (29.7 vs. 29.9 kg/m^2^; P = 0.225) and elevated blood pressure/hypertension prevalence (58.8% vs. 60%; P = 0.319) did not differ by sex. Summary characteristics of the overall analytic sample are presented in Supplementary Table S3.

**TABLE 1:** Characteristics of the Health and Retirement Study (HRS) analytic sample overall and stratified by sex (N = 8,970).

​	Female		Male	
Characteristic	n	Mean (SE)^a^	n	Mean (SE)^a^	**P** ^b^
Age, years	5,159	69.0 (0.29)	3,811	67.9 (0.27)	<0.001
Body-mass index, kg/m^2^	5,159	29.7 (0.16)	3,811	29.9 (0.10)	0.220
eGFR, ml/min/1.73 m^2^	5,159	74.5 (0.49)	3,811	77.6 (0.46)	<0.001

​	n	% (95% CI)^a^	n	% (95% CI)^a^	**P** ^b^
Race/ethnicity, self-report					0.066
Non-Hispanic White/Caucasian	3,320	77.1 (74.0, 80.1)	2,553	78.9 (75.7, 82.2)	​
Non-Hispanic Black/African American	975	10.5 (9.2, 11.9)	592	9.3 (7.9, 10.8)	​
Hispanic	714	9.4 (6.6, 12.3)	539	8.5 (5.7, 11.3)	​
Others	150	3.0 (2.3, 3.7)	127	3.2 (2.6, 3.9)	​
Education, self-report					<0.001
<Highschool	899	13.3 (11.3, 15.4)	626	11.7 (9.9, 13.4)	​
Highschool/GED	1,753	32.4 (30.6, 34.1)	1,188	29.3 (26.8, 31.9)	​
Some college	1,411	28.1 (26.4, 29.8)	906	24.2 (22.4, 26.0)	​
College/Professional degree	1,096	26.3 (24.0, 28.6)	1,091	34.7 (31.6, 37.8)	​
Alcohol use, self-report					<0.001
No	2,559	44.9 (42.4, 47.4)	1,429	33.9 (31.9, 36.0)	​
Yes	2,600	55.1 (52.6, 57.6)	2,382	66.1 (64.0, 68.1)	​
Smoking status, self-report					<0.001
Never smoker	2,658	51.3 (49.3, 53.3)	1,347	37.6 (35.7, 39.6)	​
Former smoker	1,975	38.7 (36.9, 40.4)	2,010	50.0 (47.8, 52.2)	​
Current smoker	526	10.0 (8.9, 11.1)	454	12.4 (11.0, 13.8)	​
Diabetes status					0.012
Normal	1,969	42.7 (40.7, 44.7)	1,283	38.5 (36.3, 40.7)	​
Pre-diabetic	1,533	29.6 (28.0, 31.2)	1,173	31.4 (29.2, 33.5)	​
Diabetic	1,657	27.7 (25.8, 29.7)	1,355	30.1 (28.1, 32.0)	​
Blood pressure status					<0.001
Normal	2,408	48.8 (47.4, 50.2)	1,543	40.2 (38.2, 42.1)	​
Elevated blood pressure/hypertension	2,751	51.2 (49.8, 52.6)	2,268	59.8 (57.9, 61.8)	​
Cytomegalovirus status					<0.001
Negative	1,282	31.3 (29.2, 33.4)	1,366	42.7 (39.9, 45.6)	​
Positive	3,877	68.7 (66.6, 70.8)	2,445	57.3 (54.4, 60.1)	​
eGFR binary (mL/min/1.73 m^2^)					<0.001
eGFR ≥60 mL/min/1.73 m^2^	3,754	76.0 (74.3, 77.7)	2,888	80.9 (79.2, 82.7)	​
eGFR <60 mL/min/1.73 m^2^	1,405	24.0 (22.3, 25.7)	923	19.1 (17.3, 20.8)	​

Abbreviations: n, unweighted analytic sample size; SE, weighted standard error; %, weighted proportion; 95% CI, weighted 95 percent confidence intervals; eGFR, estimated glomerular filtrate rate.

^a^ Survey weighted with Health and Retirement Study 2016 Venous Blood Study sampling weight (PVBSWGTR); b P indicates differences by sex (female versus male).

Bolded values indicate significant differences by sex.

Table 2 presents survey-weighted, adjusted geometric means (GM, 95% CI) for immune cell subsets stratified by sex (all P < 0.001 unless noted). Females generally had significantly higher geometric mean (GM) proportions in nearly half of the adaptive immune cell subsets (8 out of 17), whereas males had higher GM proportions in only 7 subsets. Specifically, females had higher GM proportions for total T cells (GM 0.70 vs. 0.67), CD4^+^ T cells (0.69 vs. 0.64), CD4^+^ naïve T cells (0.43 vs. 0.38), CD8^+^ naïve T cells (0.21 vs. 0.15), Ratio of CD4^+^ to CD8^+^ T cells (3.54 vs. 2.88), B cells (0.06 vs. 0.05), naïve B cells (0.63 vs. 0.60), and lymphocytes (0.29 vs. 0.27). Males had higher GM proportions for CD4^+^ central memory (CM; 0.36 vs. 0.32), CD4^+^ effector memory (Tem; 0.008 vs. 0.007), CD8^+^ T cells (0.24 vs. 0.22), CD8^+^ CM; 0.08 vs. 0.07), CD8^+^ effector T cell (Teff; 0.42 vs. 0.38), CD8^+^ T effector memory (Tem; 0.009 vs. 0.007), and IgD + memory B cells (0.101 vs. 0.096). No sex differences were observed for CD4^+^ Teff (P = 0.535), or IgD-memory B cells (P = 0.068). GM (95% CI) for the overall analytic sample are presented in Supplementary Table S4.

**TABLE 2:** Survey weighted adjusted geometric means (95% confidence intervals) of immune cell subsets stratified by sex.

​	Female		Male	
	n	GM (95% CI)^a^	n	GM (95% CI)^a^	**P** ^b^
T Cells	4,924	0.70 (0.69, 0.70)	3,626	0.67 (0.66, 0.68)	**<0.001**
CD4^+^ T cells	4,924	0.69 (0.69, 0.70)	3,625	0.64 (0.64, 0.65)	**<0.001**
CD4^+^ T cells: Central memory (CM)	4,896	0.32 (0.32, 0.33)	3,604	0.36 (0.35, 0.36)	**<0.001**
CD4^+^ T cells: Effector (Teff)	4,893	0.02 (0.02, 0.02)	3,597	0.02 (0.02, 0.02)	0.535
CD4^+^ T cells: Effector memory (Tem)	2,152	0.007 (0.006, 0.007)	1,624	0.008 (0.007, 0.008)	**0.017**
CD4^+^ T cells: Naïve	4,898	0.43 (0.43, 0.44)	3,597	0.38 (0.37, 0.38)	**<0.001**
CD8^+^ T cells	4,924	0.21 (0.20, 0.21)	3,626	0.24 (0.23, 0.24)	**<0.001**
CD8^+^ T cells: Central memory (CM)	3,919	0.07 (0.07, 0.07)	2,883	0.08 (0.07, 0.08)	**0.001**
CD8^+^ T cells: Effector (Teff)	4,893	0.38 (0.37, 0.39)	3,598	0.42 (0.41, 0.43)	**<0.001**
CD8^+^ T cells: Effector memory (Tem)	3,645	0.007 (0.006, 0.007)	2,645	0.009 (0.008, 0.010)	**<0.001**
CD8^+^ T cells: Naïve	4,924	3.54 (3.45, 3.63)	3,625	2.88 (2.80, 2.96)	**<0.001**
Ratio of CD4^+^ T cells to CD8+T cells	4,898	0.21 (0.21, 0.22)	3,598	0.15 (0.14, 0.15)	**<0.001**
B cells	4,924	0.060 (0.059, 0.061)	3,625	0.052 (0.051, 0.054)	**<0.001**
IgD + memory B cells	4,911	0.10 (0.09, 0.10)	3,613	0.10 (0.10, 0.10)	**0.004**
IgD- memory B cells	4,911	0.08 (0.08, 0.09)	3,614	0.09 (0.08, 0.09)	0.068
Naive B cells	4,913	0.63 (0.62, 0.63)	3,615	0.60 (0.59, 0.61)	**0.003**
Lymphocytes	5,056	0.29 (0.29, 0.30)	3,742	0.27 (0.27, 0.27)	**<0.001**

Footnote: Bolded values indicate significant differences by sex.

Abbreviations: n, Analytic sample size; GM, geometric mean; CI; 95% confidence intervals.

^a^ Survey weighted with Health and Retirement Study 2016 Venous Blood Study sampling weight (PVBSWGTR) and adjusted for age, race/ethnicity, body mass index (kg/m2), education, smoking status, alcohol use, hypertension, diabetes, and cytomegalovirus (CMV) serostatus).

^b^ P value from survey-weighted Wald test comparing females versus males.

**FIGURE 1:**
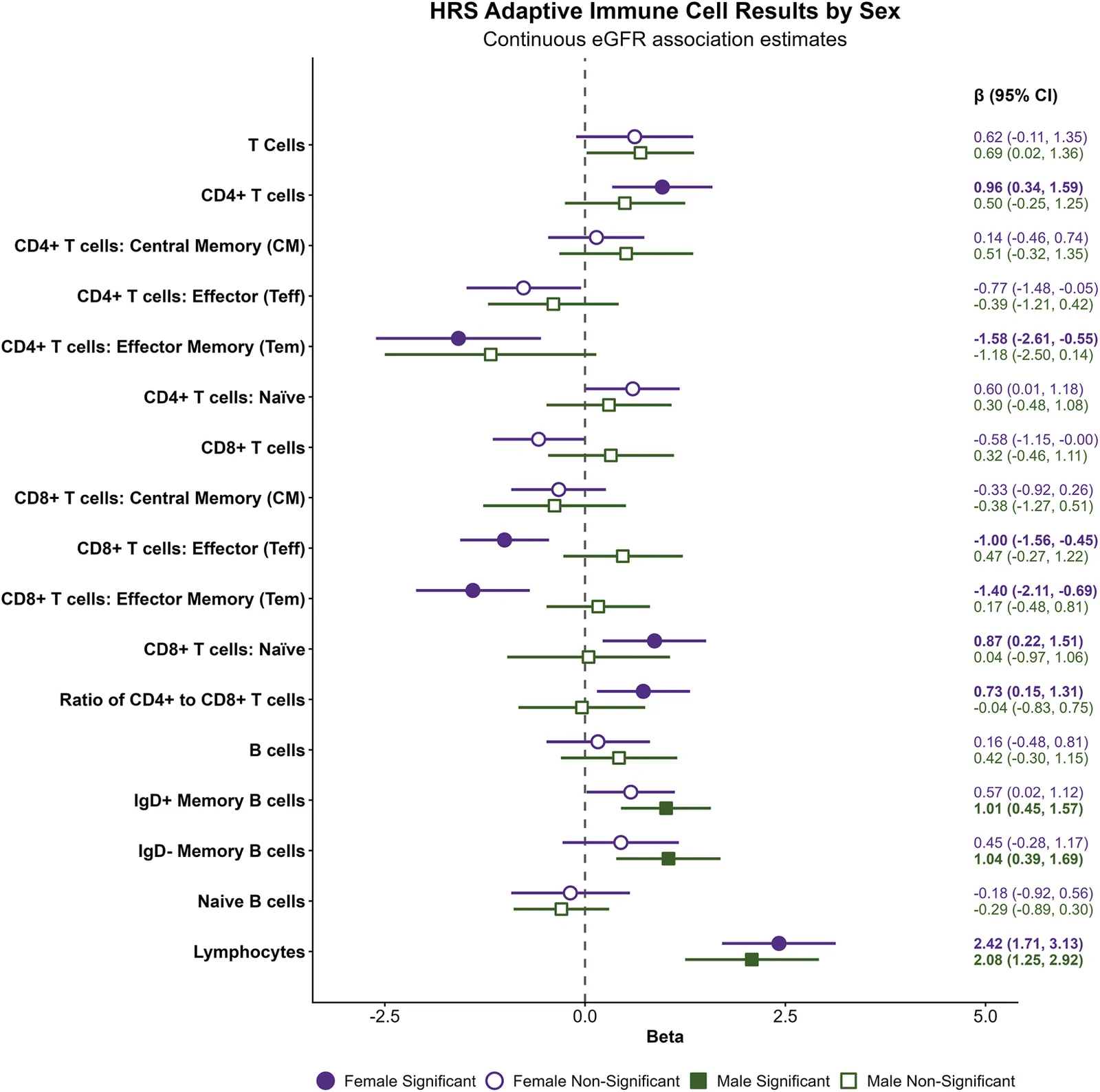
Sex-stratified associations between adaptive immune cell subset proportions and estimated glomerular filtration rate (eGFR, mL/min/1.73 m^2^). βeta coefficients and 95% confidence intervals (CI) represent the change in eGFR per one-SD increase in log-transformed immune cell proportion, adjusted for age at blood collection, race/ethnicity, body mass index (BMI), smoking status, alcohol use, blood pressure status, diabetes status, and cytomegalovirus (CMV) serostatus. Analyses were conducted separately by sex (female, male) and sample sizes can be found in Supplementary Table S2. Significance was based on FDR-adjusted p-values and calculated using the Benjamini–Hochberg method (Benjamini and Hochberg, 1995). Dark shading indicates statistically significant associations (P_FDR_<0.05); light shading indicates non-significant associations P_FDR_ ≥ 0.05). Abbreviations: CM, central memory; Teff, effector; Tem, effector memory.

**FIGURE 2:**
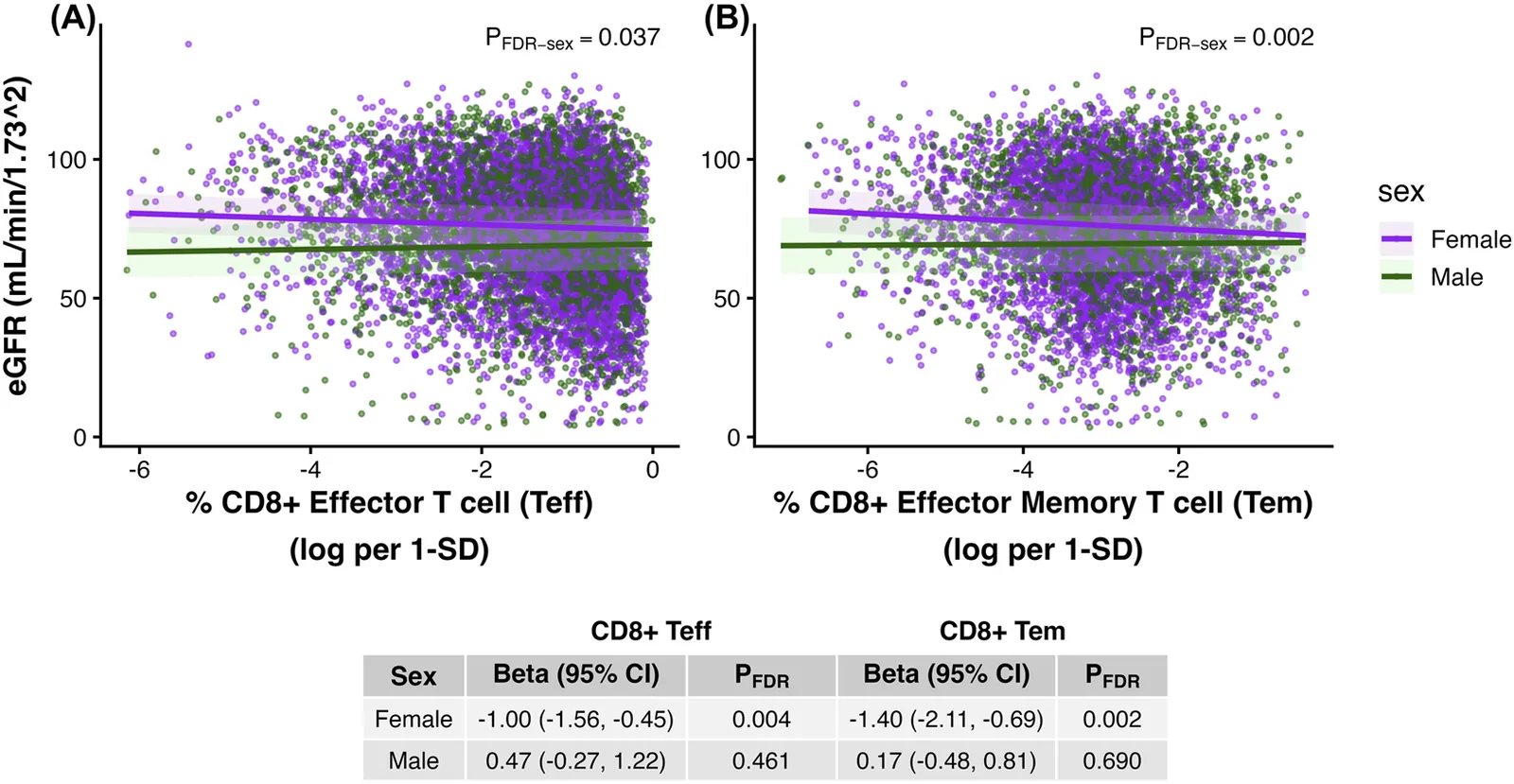
Adjusted association between **(A)** CD8^+^ effector T cell (Teff) and **(B)** CD8^+^ effector memory T cell (Tem) proportions (%) with estimated glomerular filtration rate (eGFR, mL/min/1.73 m^2^), stratified by sex (female, male). Lines represent stratified linear regression models adjusted for age at blood collection, race/ethnicity, education, body-mass index (BMI), smoking status, alcohol use, blood pressure status, diabetes status, and cytomegalovirus (CMV) serostatus. FDR-adjusted p-values were calculated using the Benjamini–Hochberg method (Benjamini and Hochberg, 1995). Statistically significant associations are based on P_FDR_<0.05. Abbreviations: Teff, effector; Tem, effector memory.

Sex-stratified analyses (Figure 1; Supplementary Figure S2; Supplementary Table S5) revealed sex differences in adaptive immune cell subset associations with eGFR. In females, higher proportions of CD4^+^ T cells, CD8^+^ naïve T cells, IgD + memory B cells, and lymphocytes were associated with higher eGFR (higher kidney function), while higher CD4^+^ Teff, CD4^+^ Tem, CD8^+^ T cells, CD8^+^ Teff, and CD8^+^ Tem were associated with lower eGFR (lower kidney function). In males, higher proportions of T cells, IgD+ and IgD-memory B cells, and lymphocytes were associated with higher eGFR (higher kidney function). Notably, there were significant sex interactions for CD8^+^ Tem (P_FDR_ = 0.015) and nominally significant for CD8^+^ Teff (P_FDR_ = 0.060) in the overall model (Supplementary Table S7). For example, in females a one-SD increase in log-transformed CD8^+^ Teff was associated with 1.00 mL/min/1.73 m^2^ lower eGFR (β = −1.00 per SD, 95% CI = −1.56, −0.45, P_FDR_ = 0.004), and in CD8^+^ Tem with 1.40 mL/min/1.73 m^2^ lower eGFR (β = −1.40, 95% CI = −2.11, −0.69, P_FDR_ = 0.002), whereas effects were null in males (β_CD8+ Teff_ = 0.47, 95% CI = −0.27, 1.22, P_FDR_ = 0.461 and β_CD8+ Tem_ = 0.17, 95% CI = −0.48, 0.81, P_FDR_ = 0.690; Figure 2). Figure 2 illustrates the divergent sex-specific associations for CD8^+^ Teff and Tem cell proportions with eGFR, with inverse associations observed among females and null-to-positive associations among males. Supplementary Figure S2 presents a heatmap of beta coefficients across all 17 adaptive immune cell subsets, contextualizing these findings within the broader pattern of sex-stratified associations. Given that CRP and related cytokines may lie downstream of immune cell activation, we conducted a sensitivity analysis additionally adjusting for an inflammatory latent class variable comprising CRP, IL-6, IL-10, IL-1RA, and TNFR1 (Supplementary Table S5). Among females, associations for CD8^+^ Teff and CD8^+^ Tem were attenuated but remained directionally consistent and nominally significant after inflammatory adjustment (β_CD8+ Teff_ = −0.68, 95% CI = −1.17, −0.19, P_FDR_ = 0.060 and β_CD8+ Tem_ = −0.98, 95% CI = −1.64, −0.32, P_FDR_ = 0.060, respectively), suggesting these associations are not fully explained by systemic inflammation. Among males, associations remained null to modestly positive after inflammatory adjustment (β_CD8+ Teff_ = 0.63, 95% CI = −0.07, 1.33, P_FDR_ = 0.436 and β_CD8+ Tem_ = 0.60, 95% CI = 0.02, 1.17, P_FDR_ = 0.349). Sensitivity analysis restricted to participants with eGFR ≥60 mL/min/1.73 m^2^ and eGFR ≥30 mL/min/1.73 m^2^ (Supplementary Table S6) demonstrated consistent, albeit attenuated, patterns among females for CD8^+^ Teff (β = −0.70, 95% CI = −1.27, −0.13, P_FDR_ = 0.091 and β = −0.74, 95% CI = −1.27, −0.22, P_FDR_ = 0.034, respectively) and CD8^+^ Tem (β = −0.85, 95% CI = −1.37, −0.33, P_FDR_ = 0.034 and β = −1.29, 95% CI = −2.03, −0.56, P_FDR_ = 0.007, respectively). This suggests that sex-specific immune kidney associations are present even among those with preserved or mildly reduced kidney function, extending beyond moderate-to-severe kidney function. Given CMV seroprevalence differences by sex (68.7% females vs. 57.3% males, P < 0.001; Table 1) and CMV’s role in driving CD8^+^ Teff/Tem expansion, we tested for effect modification by CMV on the immune-eGFR associations within each sex stratum; no significant interaction was observed (all CMV interaction P_FDR_>0.360; Supplementary Table S5). In additional sensitivity analyses restricted to the subset of females with confirmed post-menopausal status, associations between CD8^+^ Teff/Tem T-cell subsets, IgD^+^ memory B cells, lymphocytes, and eGFR were similar in direction and magnitude to those observed in the full female sample (Supplementary Table S7), supporting the robustness of the sex-specific immune cell and kidney associations within a predominantly post-menopausal population.

In the overall sample, associations between adaptive immune cell subsets and eGFR were modest and not uniformly consistent across cell types (Supplementary Table S8). Higher proportions of log-transformed total T cells, CD4^+^ T cells, CD4^+^ naïve T cells, IgD+ and IgD-memory B cells, and lymphocytes were associated with higher eGFR (higher kidney function), while higher proportions of CD4^+^ Tem and CD8^+^ Tem were associated with lower eGFR (lower kidney function). For example, a one-SD increase in log-transformed lymphocyte proportion was associated with a 2.29 mL/min/1.73 m^2^ higher eGFR (β = 2.29, 95% CI 1.77, 2.81; P_FDR_<0.001), while a one-SD increase in log-transformed CD4^+^ Tem proportion was associated with 1.37 mL/min/1.73 m^2^ lower eGFR (β = −1.37, 95% CI = −2.13, −0.60; P_FDR_ = 0.004). After additional adjustment for the inflammation latent class variable (Supplementary Table S8), most associations were attenuated toward the null (e.g., β = −0.33, 95% CI = −0.75, 0.08, P_FDR_ = 0.464 for lymphocytes and β = −0.79, 95% CI = −1.51, −0.07, P_FDR_ = 0.391 for CD4^+^ Tem), suggesting that part of the signal may be mediated by systemic inflammation. The sensitivity analysis restricted to participants with eGFR ≥60 mL/min/1.73 m^2^ and eGFR ≥30 mL/min/1.73 m^2^ (Supplementary Table S9) demonstrated directionally consistent associations for lymphocytes and IgD+/IgD-memory B cells, with memory B cell effects remaining robust and, in some cases, slightly stronger across both restricted samples, whereas lymphocyte and CD4^+^ and CD8^+^ Tem cell associations were attenuated in the preserved kidney function group (eGFR ≥ 60) but persisted among participants with eGFR ≥ 30 mL/min/1.73 m^2^. This pattern, consistent with findings in the sex-stratified analyses, suggests that immune cell associations with kidney function, including the expansion of Teff and Tem T cell populations and shifts in lymphocyte and memory B cells, are evident and may begin to emerge before more advanced reductions in eGFR.

## Discussion

Our cross-sectional analysis demonstrates distinct, sex-specific associations between adaptive immune cells and kidney function in adults aged ≥ 56 years. The most notable divergence involved CD8^+^ effector (Teff) and effector memory (Tem) T cell subsets: in females, higher CD8^+^ Teff and Tem (both hallmark indicators of T cell differentiation and immune aging), along with CD4^+^ Tem, were significantly associated with lower eGFR (lower kidney function), whereas in males these same CD8^+^ subsets demonstrated null-to-modestly positive associations with eGFR (higher kidney function) that were not FDR-significant. Conversely, higher proportions of CD4^+^ T cells, naïve CD8^+^ T cells, the ratio of CD4^+^ to CD8^+^ T cells, and lymphocytes were associated with higher eGFR in females, suggesting that immune profiles enriched with naïve and helper T cell populations reflect a “younger” immune profile and may correlate with better kidney function. The magnitude of these associations is modest but biologically relevant: in females, a one-SD increase in CD8^+^ Teff or Tem was associated with roughly 1–1.5 mL/min/1.73 m^2^ lower eGFR, compared to approximately 1–2 years of age-related decline in kidney function in older adults. In males, a distinct pattern emerged where memory B cell subsets (IgD+ and IgD-) and lymphocytes were associated with higher eGFR. Taken together, these findings suggest that cross-sectional associations between immune phenotypes and kidney function differ by sex, raising the hypothesis that females may be more vulnerable to T cell associated “inflammaging” in the context of renal aging; however, causal pathways or temporal ordering cannot be determined from these cross-sectional data.

Terminally differentiated CD8^+^ T cells lose their proliferative capacity but gain pro-inflammatory and cytotoxic functions (Litjens et al., 2024; Layug et al., 2024). In the aging kidney, these cells are known to infiltrate the renal interstitium (connective tissue compartment of the kidney) and secrete high levels of interferon gamma (IFNγ) and tumor necrosis factor (TNFα) to promote microvascular damage and fibrosis (Be et al., 2012; Leonhard et al., 2022; Jiang et al., 2024). In our study, senescent-like CD8^+^ Teff and Tem subsets were inversely associated with eGFR in older females, independent of CMV serostatus, suggesting that expansion of differentiated CD8^+^ T cell population co-occur with lower kidney function in this group. This pattern is consistent with broader evidence of heterologous antigen stimulation and bi-directional kidney-immune interplay that intensifies with age (Espi et al., 2020; Chi et al., 2022). However, because our analyses are cross-sectional, we cannot determine whether these CD8^+^ T cell changes precede kidney function decline or arise as a consequence of CKD-associated immunosenescence. Our results in an older ( ≥ 56 years) cohort align with prior work showing strong age-related expansion of CD8^+^ Teff and Tem subsets that are largely independent of CMV, and they also parallel ESRD studies in which highly differentiated CD8^+^ T cells have been linked to cardiovascular and cardio-renal events (Be et al., 2012; Betjes et al., 2011; Thyagarajan et al., 2022; Leonhard et al., 2022). Together, these data suggests that CD8^+^ T cell differentiation may reflect a continuum of age- and disease-related immune remodeling rather than a single disease-specific phenotype, which our cross-sectional study can describe not mechanistically separate. Women typically show slower CKD progression before menopause (despite a higher prevalence), partly due to metabolic programming and lower *EGFR* expression (Liao et al., 2026; Klein and Flanagan, 2016; Kattah and Garovic, 2020; Melsom et al., 2022). In the context of our older, predominately postmenopausal population, our findings suggest that sex-biased immune aging and renal aging remain closely associated in females; however, whether loss of hormonal protection directly modifies these associations will require longitudinal and mechanistic studies.

We hypothesize that the observed sex divergence in CD8^+^ Teff and Tem associations with eGFR may reflect differences in hormonal regulation of T cell-mediated inflammation between older females and males, such that similar CD8^+^ effector/memory reservoirs could be subject to distinct regulatory constraints in different sex-specific hormonal environments. In females, the postmenopausal withdrawal of estradiol (E2), which has been proposed to act as a “molecular brake” on T cell-mediated inflammation, could plausibly unmask immune-mediated renal injury (Wu et al., 2021; Xing et al., 2012). Menopause status was unavailable for most participants; however, among females with available data (n = 2,785), nearly all (98.9%) self-reported being postmenopausal. Sensitivity analysis restricted to confirmed postmenopausal females demonstrated findings similar to those in the full female sample, supporting the relevance of a pre-dominantly postmenopausal context while not establishing hormonal mechanisms (Supplementary Table S7). Experimental and clinical data suggest that loss of E2 can increase T cell activation and effector function, potentially enhancing CD8^+^ T cell cytotoxic activity (Layug et al., 2024; Ghosh et al., 2014; Sciarra et al., 2023); in our study, we interpret this literature as providing biological plausibility rather than evidence of causation, and we cannot distinguish whether CD8^+^ Teff/Tem composition serves primarily as a biomarker of renal risk, of hormonal changes, or both. Conversely, older males may maintain a more stable or functionally “exhausted” or “spent” CD8^+^ T cell profile, influenced in part by the androgen-androgen receptor signaling that has been linked to reduced cytokine output despite high memory T cell burden (Bader et al., 2025; Ning et al., 2023). Consequently, the accumulated CD8^+^ Teff reservoir in males may be less likely to translate into the same degree of renal damage observed in postmenopausal females, though this interpretation remains speculative and requires prospective validation.

Higher CD4^+^ T cells, naïve CD8^+^ T cells, and the ratio of CD4^+^ to CD8^+^ T cells were significantly associated with higher eGFR (higher kidney function) in females. This positive association suggests that immune profiles enriched with naïve cells and CD4^+^ helper populations tend to co-occur with better kidney function in this group, consistent with a “younger” adaptive immune profile in older adults (Chen et al., 2025; Thyagarajan et al., 2022). In aging populations, the inversion of the CD4/CD8 ratio (due to CD8^+^ memory expansion) is a robust predictor of morbidity and systemic “inflammaging” (Márquez et al., 2020; Gheitasi et al., 2025). Our results are in line with the idea that the shift from naïve to memory dominance appears to be a critical tipping point for renal health in aging females, rather than proving that immune aging directly causes kidney decline. CD4^+^ Th1 and Th17 subsets typically provide the necessary “help” for CD8^+^ activation and expansion, but the CD4^+^ compartment also includes regulatory T cells (Tregs) that are essential for suppressing inflammation. In postmenopausal females, a preserved CD4^+^ T cell population may help counterbalance the chronic inflammatory state associated with expanded CD8^+^ memory subsets. This potential protective role is often limited by immunosenescence, as aging CD4^+^ T cells typically exhibit reduced naïve pools, expansion of exhausted phenotypes, and restricted TCR diversity. Overall, our findings align with suggestions that a robust, “younger” CD4^+^ and naïve CD8^+^ T cell profile supports renal filtration, whereas the transition toward immunosenescence and exhaustion is associated with low-grade inflammation and microvascular damage, although causal relationships cannot be inferred from our cross-sectional data (Hartzell et al., 2020).

Interestingly, B cell associations were also sex specific, with positive associations with IgD+ and IgD-memory B cell subsets in males, whereas in females the association for IgD + memory B cells was weak and the association for IgD− memory B cells was null. Memory B cells can produce regulatory cytokines such as IL-10 and can be both pro-inflammatory and contribute to immune homeostasis (Veh et al., 2024). In our data, higher memory B cell proportions in older males ( ≥ 56 years) were associated with better kidney function, raising the possibility that memory B cell-mediated regulatory pathways may be more prominent in males than in females in this age range. One speculative explanation is that estrogen-dependent class-switched memory B cell declines observed postmenopause could reduce B cell-mediated regulatory capacity in females, whereas males may retain or expand memory B cell pools that support renal homeostasis (Peckham et al., 2025). This interpretation aligns with reports of declining B memory and follicular B cells in females across adulthood (starting at ∼27 toward later life ∼93 years old), linked to estrogen fall and retention of naïve B cell populations (Gheitasi et al., 2025). However, because B cell function and hormone levels were not directly measured in this study, we treat these sex-specific B cell hypotheses as correlative and do not infer that B cells themselves are protective in a causal sense. Overall, these findings highlight the adaptive immune patterns associated with kidney function are not uniform across sexes where females may be more vulnerable to T cell-mediated damage and males may rely relatively more on B cell mediated regulatory pathways to maintain glomerular filtration, a possibility that warrants testing in mechanistic and longitudinal studies (Khan et al., 2026).

Most participants in our analytic sample had preserved or mildly reduced kidney function (eGFR ≥60 mL/min/1.73 m^2^), and sensitivity analyses restricted to eGFR ≥60 and ≥30 mL/min/1.73 m^2^ yielded patterns similar to the full sample. This suggests that the observed associations between immune cells and kidney function, particularly the expansion of CD8^+^ Tem and Teff T cell subsets in females, may manifest early in the trajectory of kidney function decline, before more severe impairment. These patterns contrast with advanced CKD and ESRD cohorts, where more extreme naïve T-cell depletion and CD28^−^ expansions predominate, and our findings may therefore capture an earlier phase of immunosenescence along the cardio-renal axis (Chi et al., 2022; Winterberg and Ford, 2017; Betjes et al., 2011; Leonhard et al., 2022). As this study is cross-sectional, we cannot determine whether these immune changes precede or result from early declines in kidney function.

There are several strengths of our analyses. First, this study leverages multiparameter flow cytometry, the “gold standard” method for immunophenotyping, to precisely quantify adaptive immune cell subsets (naïve, effector memory, and terminally differentiated T and B cells). Second, the HRS 2016 VBS provides a large, nationally representative U.S. sample, ensuring that our findings regarding sex-specific immune profiles are generalizable to the aging U.S. population. Finally, our multivariable adjustment for primary clinical risk factors for renal decline (e.g., elevated blood pressure/hypertension, diabetes, obesity) demonstrated that these immune-eGFR associations exist independent of traditional metabolic and vascular risk factors.

However, several limitations warrant mention. First, the cross-sectional nature of this analysis precludes the establishment of a temporal or causal relationship between T-cell differentiation and kidney function. Second, despite adjustment for major clinical and sociodemographic covariates, residual confounding cannot be excluded. Variables unavailable in the 2016 HRS venous blood sample including albuminuria, detailed medication use and reasons for use, and objective physical activity, as well as potentially mediating inflammatory biomarkers, may influence both immune cell composition and kidney function; their absence should temper causal interpretation of our findings. Third, the lack of information on hormone replacement therapy (HRT) and specific timing of the menopause transition status (such as years since last menses) limits our ability to see how estrogen plays a role in the relationship between immune cells and kidney function. Finally, single-point blood measurement may not capture the long-term immune cell profile of the participants as effectively as longitudinal sampling, as immune cells can fluctuate due to acute, transient infections. Future research should utilize longitudinal data to determine whether accelerated T cell differentiation precedes eGFR decline or if it is a consequence of pro-inflammatory environment characteristics of CKD. Additionally, studies incorporating prospective hormonal assays and HRT history would further clarify the mechanisms by which sex hormones modulate T cell-mediated renal injury.

In conclusion, these findings suggest that CD+8 Tem and Teff T cells could serve as sex-specific biomarkers for renal risk in postmenopausal females. These results are hypothesis-generating and do not establish that CD8^+^ T cell differentiation causally drives renal decline. Therapeutic strategies aimed at modulating immune aging, such as approaches to reduce senescent T cell burden or mimic estrogenic anti-inflammatory effects, have been proposed in other contexts and may warrant investigation as potential avenues for preserving eGFR in females, but such interventions will require rigorous testing in longitudinal and mechanistic studies before their relevance to kidney disease can be determined.

## Data Availability

The results in this study were generated through the analysis of sensitive health data from the Health and Retirement Study (HRS), which could be obtained through a sensitive data use agreement with the HRS at https://hrsdata.isr.umich.edu/.

## Supplementary material

SUPPLEMENTARY FIGURE S1 Flow diagram of participant inclusion and exclusion for the cross-sectional analysis of associations between adaptive immune cell subsets and kidney function in the 2016 Health and Retirement Study Venous Blood Study.

SUPPLEMENTARY FIGURE S2 Heatmap of sex-stratified associations between adaptive immune cell subsets and estimated glomerular filtration rate (eGFR, mL/min/1.73 m2). Beta coefficients from sex-stratified survey-weighted linear regression models examining associations between adaptive immune cell subsets and continuous eGFR (mL/min/1.73m^2^) among older adults in the Health and Retirement Study (2016). Models adjusted for age, race/ethnicity, education, body-mass index (BMI), smoking status, alcohol use, hypertension, diabetes, and cytomegalovirus (CMV) serostatus. Blue indicates inverse associations (higher immune cell proportion associated with lower eGFR); red indicates positive associations (higher immune cell proportion associated with higher eGFR). Asterisks denote statistically significant associations based on FDR-adjusted P < 0.05 (Benjamini-Hochberg method). Abbreviations: Teff, effector T cells; Tem, effector memory T cells; CM, central memory T cells; Tn, naïve T cells; NK, natural killer cells; eGFR, estimated glomerular filtration rate; FDR, false discovery rate; CMV, cytomegalovirus.
